# A Comprehensive Study of Positive Body Image as a Predictor of Psychological Well-Being in Anorexia Nervosa

**DOI:** 10.3390/nu16111787

**Published:** 2024-06-06

**Authors:** Sandra Torres, Ana Isabel Vieira, Filipa Mucha Vieira, Kylee M. Miller, Marina Prista Guerra, Leonor Lencastre, Ana Catarina Reis, Sertório Timóteo, Patrícia Nunes, Maria Raquel Barbosa

**Affiliations:** 1 Faculty of Psychology and Education Sciences, University of Porto, 4200-135 Porto, Portugal; fvieira@fpce.up.pt (F.M.V.); mguerra@fpce.up.pt (M.P.G.); leonor@fpce.up.pt (L.L.); raquel@fpce.up.pt (M.R.B.); 2 Center for Psychology at the University of Porto (CPUP), University of Porto, 4200-135 Porto, Portugal; anavieira@fpce.up.pt; 3 Child Development and Rehabilitation Center (CDRC), Institute on Development & Disability, Oregon Health & Science University, Portland, OR 97403, USA; millerky@ohsu.edu; 4 Department of Psychiatry, Centro Hospitalar de São João, 4200-319 Porto, Portugal; ana.silva.reis@ulssjoao.min-saude.pt (A.C.R.); sertoriotimoteo@gmail.com (S.T.); patricialopesnunes@gmail.com (P.N.)

**Keywords:** anorexia nervosa, eating disorder, positive body image, body appreciation, functionality appreciation, body responsiveness, emotion regulation, psychological well-being

## Abstract

Recent data suggest a close association between positive body image (PBI) and eating disorder recovery. Nevertheless, the specific mechanisms through which PBI may facilitate recovery from anorexia nervosa (AN) remain unknown. To advance understanding of these mechanisms, this study examined core indices of PBI within AN, exploring its association with emotion regulation and well-being outcomes. Data were collected from 159 female participants, 64 with AN diagnosis and 95 healthy controls (HCs), who completed measures of PBI (body appreciation, functionality appreciation, and body responsiveness), emotion regulation, and psychological well-being (depression, anxiety, stress, and psychological quality of life). The AN group reported lower levels of PBI and psychological well-being, along with greater difficulties in regulating emotions, relative to HCs. PBI variables significantly predicted emotion regulation and psychological well-being in AN, accounting for 36% to 72% of the variance, with body appreciation emerging as the strongest predictor. These findings lend credence to the view that PBI can serve as a catalyst for psychological health. We hypothesize that enhancing PBI can improve interoceptive awareness, which is crucial for emotion regulation and reducing maladaptive food-related coping. Emphasizing a mind–body connection in lifestyle could be a relevant element to consider for both treating and preventing AN.

## 1. Introduction

Anorexia nervosa (AN) is characterized by a persistent restriction of energy intake, an intense fear of gaining weight, and body image disturbance [1]. Negative body image (NBI) is a prominent feature in AN [2,3], as individuals with AN typically exhibit a disturbance in the perception of their body size, coupled with an undue emphasis on body shape and weight in self-evaluation [1]. In comparison to individuals without eating disorders, those with AN displayed diminished accuracy in estimating their body size, higher levels of body dissatisfaction, increased negative emotions and thoughts toward the body, and a greater frequency of behaviors such as body checking [4].

According to the systematic review by Glashouwer et al. [5], NBI is associated with the trajectory of AN, encompassing its onset, persistence, and relapse. For this reason, clarifying the role of body image in AN symptomatology and its relevance as a treatment target is a research area that warrants investment. In addition, there is considerable room for further exploration in studying body image in AN, given that existing research has been dominated by a focus on negative aspects, which does not offer a comprehensive understanding of the body’s experience. Body image includes positive and negative features alongside perceptions, thoughts, and feelings about one’s body [6,7]. Positive body image (PBI) is a multidimensional construct not defined in opposition to NBI. PBI is an independent construct that involves multiple facets, including appreciation of the body and its functional abilities, body acceptance, a broad conceptualization of beauty, inner positivity, and awareness of the body’s needs [8].

In the context of AN, PBI is a research field with limited exploration. Nevertheless, some findings in the literature suggest that PBI may play a pivotal role in enhancing eating behavior. Studies examining body appreciation—a central component of PBI involving the respect and appreciation of one’s body’s features, functionality, and health [9]—have revealed that elevated levels of body appreciation negatively correlated with disordered eating behaviors among undergraduate students [10,11]. Conversely, individuals’ attunement to their hunger cues (i.e., intuitive eating) [12] was positively associated with body appreciation in female college students [13]. In samples with eating disorders, the limited existing studies suggest that improvements in body appreciation during treatment were associated with a reduction in eating symptomatology [14]. Additionally, body appreciation was found to be closely linked to eating disorder recovery status [15].

The possible mechanisms by which PBI can facilitate AN recovery are unknown. One plausible pathway is that PBI serves as a catalyst for psychological health and overall well-being. The relationship between PBI and indicators of well-being has not yet been addressed in AN; however, in non-clinical samples, this evidence is well established, in particular in what concerns depression and anxiety symptoms [11,16] and quality of life (QoL) [17,18].

From a theoretical perspective, emotion regulation may play a pivotal role in this process, acting in conjunction with PBI to facilitate a reduction in affective and AN symptoms. It has been widely recognized that individuals with AN have less emotional awareness/clarity over emotions [19], increased levels of alexithymia (e.g., [20,21,22]), and difficulties in regulating emotions (e.g., [20,23,24]) when compared to healthy controls (HC). Individuals with a lower emotional regulation capacity have a higher risk to engage in dysfunctional mood-modulatory behaviors, such as binge eating, self-induced vomiting, or excessive exercise [25,26]. These behaviors may serve as ways to control or escape from intense emotions, which contribute to the perpetuation of the disorder [27] or long-term relapse [28]. In addition, the co-occurrence of emotional disorders in AN, such as anxiety and depression, can be rooted in emotional processing impairments [29,30].

The association between emotion regulation and body experience has been established in non-clinical samples. For example, individuals with difficulty identifying emotional states presented higher body image disturbance [31]. Conversely, multiple facets of positive body image have been associated with adaptive emotion regulation skills [32] and reduced levels of alexithymia [33]. Difficulties in regulating emotions seem to be associated with a lower attunement to the body’s needs and the use of embodied information to guide behavior [34], as denoted by the concept of body responsiveness—a fundamental aspect of embodiment [35]. From an embodiment perspective (i.e., the experience of connection with the body), the accurate detection and interpretation of bodily cues facilitate access to essential mechanisms of emotion regulation. Successful emotion regulation is intricately tied to being attuned to bodily signals, known as interoceptive awareness [36,37]. By paying attention to the body’s needs, individuals are more likely to engage in adaptive self-care behaviors, an essential aspect of PBI [8].

In sum, the literature suggests that positive embodiment can benefit physical and psychological well-being [38]. Based on these findings from non-clinical samples, we hypothesized that PBI, as an adaptive construct grounded in positive embodiment [39] and affect regulation [40,41], can improve several aspects of psychological functioning in AN. To assess the viability of this hypothesis, this study aims to characterize core indices of PBI in individuals with AN, namely body appreciation, functionality appreciation, and body responsiveness, while examining their putative associations with emotion regulation and psychological well-being. We sought (1) to compare the PBI dimensions between individuals diagnosed with AN (AN group) and an HC group and (2) to investigate whether PBI predicts difficulties in emotion regulation, emotional symptoms (depression, anxiety, and stress), and psychological QoL.

Considering that AN is often understood as a body image disorder [42], it might seem intuitive to assume that the AN group would present lower levels of body appreciation [43], functionality appreciation and body responsiveness when compared to the HC group. As both PBI and effective emotion regulation require mechanisms of interoceptive awareness [33,36], we also predicted that higher scores in the PBI dimensions would correlate with fewer difficulties in emotion regulation among individuals with AN [32,33,34]. Based on the research conducted with non-clinical samples, we anticipated a positive correlation between PBI dimensions and psychological QoL [17,18,34] and a negative correlation with emotional symptomatology [11,16]. Finally, we hypothesized that the combined dimensions of the PBI would significantly predict all indicators of psychological well-being and difficulties in emotion regulation. Owing to the limited number of existing studies on AN, no specific predictions were made regarding the individual contributions of each PBI dimension in the tested models.

## 2. Materials and Methods

### 2.1. Participants

The sample was collected during the years 2021 and 2022 and consisted of 159 women, 64 (40.3%) who had diagnoses of AN (*Mage* = 25.75, *SD* = 9.63, range 16–55 years), and 95 (59.7%) composed the HC group (*Mage* = 26.80, *SD* = 8.20, range 16–50 years). There were no significant group differences in age (*t* (157) = −0.74, *p* = 0.46, *d* = −0.12).

For the AN group, the eligibility criteria were being 16 years or older and having a medical diagnosis of AN restricting type or binge eating/purging type, according to the Diagnostic and Statistical Manual of Mental Disorders 5th edition (DSM-5) [44]. The inclusion criterion for the HC group was 16 years or older and normal weight based on weight status categories defined by the World Health Organization (WHO, 2010; body mass index—BMI—between 18.5 and 24.9 kg/m^2^). In both groups, participants were excluded if they met one of the following self-reported criteria: current pregnancy or lactation, gender dysphoria, self-harm behaviors, and concurrent psychiatric or medical disorders.

There were no significant differences between the AN group (*M* = 13.30, *SD* = 2.91) and the HC group (*M* = 14.02, *SD* = 2.55) in years of schooling (*t* (157) = −1.66, *p* = 0.10, *d* = −0.27). The HC group’s mean BMI was 21.59 kg/m^2^ (*SD* = 1.89), ranging from 18.59 to 24.44 kg/m^2^. The BMI in the AN group ranged from 12.19 to 18.49 kg/m^2^ (*M* = 15.59 kg/m^2^; *SD* = 1.71). The mean AN duration was 57.83 months (*SD* = 76.80), ranging from just diagnosed to 300 months post-diagnosis. The duration of the disorder was not significantly associated with PBI (.01 < *r* < 0.22; *p* > 0.08) or psychological well-being variables (−0.01 < *r* < −0.16; *p* < 0.27).

### 2.2. Material

#### 2.2.1. Demographics

Participants completed a questionnaire that collected information on age, years of schooling, weight, height, and disease duration (the latter only in the AN group).

#### 2.2.2. Body Appreciation

Body appreciation was assessed by the Body Appreciation Scale-2 (BAS-2) [17,45,46]. The BAS-2 comprises 10 items (e.g., “I respect my body”), which assess acceptance, respect, and care for one’s body and protection of one’s body from unrealistic beauty standards. All items were rated on a 5-point Likert-type scale, ranging from 1 (never) to 5 (always). The total score was computed as the mean of all items, with the higher scores reflecting greater body appreciation. In the present study, Cronbach’s α was 0.93 for both the AN and HC groups.

#### 2.2.3. Functionality Appreciation

Functionality appreciation was assessed using the Functionality Appreciation Scale (FAS) [47,48], which examines participants’ attitudes towards body functionality, including appreciation, respect, and honoring the body’s capabilities. The FAS is composed of seven items (e.g., “I appreciate my body for what it is capable of doing”) rated on a 5-point Likert-type scale, ranging from 1 (strongly disagree) to 5 (strongly agree). The total score was computed as the mean of all items, with the higher scores reflecting a greater appreciation of body functionality. In the present study, Cronbach’s α was 0.86 for both the AN and HC groups.

#### 2.2.4. Body Responsiveness

Participants completed the Body Responsiveness Questionnaire (BRQ) [34,35]. The BRQ evaluates the tendency to be attuned to the body’s needs and use embodied information to guide behavior. This is a 7-item self-reported measure rated on a 7-point Likert-type scale, ranging from 1 (not at all true about me) to 7 (very true about me) across two factors: (1) importance of interoceptive awareness (i.e., BRQ-Importance), which assesses the importance of using interoceptive information to guide behavior (e.g., “I am confident that my body will let me know what is good for me”), and (2) perceived connection (i.e., BRQ-Perceived Connection), which assesses the extent of the (dis)connection between body and mind (e.g., “My mind and my body often want to do different things”). The total score was computed as the mean of all items, with higher scores reflecting greater body responsiveness. In this study, Cronbach’s α for the BRQ-Importance factor was 0.86 in the AN group and 0.91 in the HC group. The BRQ-Perceived Connection factor presented lower internal consistency, with an alpha of 0.63 in the AN group and 0.68 in the HC group.

#### 2.2.5. Difficulties in Emotion Regulation

The Difficulties in Emotion Regulation Scale (DERS) [49,50] assessed difficulties in emotion regulation. This is a 36-item self-report measure rated on a 5-point Likert-type scale, ranging from 1 (almost never) to 5 (almost always), across six dimensions: (1) limited access to emotion regulation strategies, (2) non-acceptance of emotional responses, (3) lack of emotional awareness, (4) impulse control difficulties, (5) difficulties engaging in goal-directed behavior, and (6) lack of emotional clarity. Although it is possible to obtain a total score (sum of all items) and a score for each dimension, in this study, we used only the total score. Higher total scores indicate more significant difficulties in emotion regulation. In the present study, Cronbach’s α was 0.96 for the AN group and 0.94 for the HC group.

#### 2.2.6. Depression, Anxiety, and Stress Symptoms

Participants answered the 21-item Depression, Anxiety and Stress Scale (DASS) [51,52]. This scale assesses depression, anxiety, and stress symptoms. Participants were asked to refer to the previous week and provide their answers on a 4-point Likert-type scale, ranging from 0 (did not apply to me at all) to 3 (applied to me very much, or most of the time). Items are summed for each subscale, with higher scores indicating more symptoms. In the AN group, Cronbach’s α for the subscales varied between 0.90 and 0.94; in the HC group, it varied between 80 and 0.88.

#### 2.2.7. Psychological QoL

The WHO Quality of Life Scale Abbreviated Version (WHOQOL-Bref) [53,54] is a 26-item self-report questionnaire assessing four areas of QoL: (1) physical, (2) psychological, (3) social, and (4) environment. Only items comprising the psychological domain were applied in the present study. Participants rated items on a 5-point Likert-type scale that measured intensity, frequency, evaluation, and capacity. Higher scores are indicative of higher psychological QoL. In the present study, Cronbach’s α was 0.90 for the AN group and 0.83 for the HC group.

### 2.3. Procedure

Participants from the AN group were recruited from a public psychiatric service that provides specialized treatment for eating disorders in the north of Portugal. All participants were in outpatient treatment, were referred by clinicians, and completed paper-and-pencil questionnaires. Psychiatrists diagnosed participants with AN according to the DSM-5 [44] criteria. Criterion A, which requires that the individual’s weight be significantly low, was operationalized as a BMI below 17 kg/m^2^. According to DSM-5, this criterion was also validated in participants with a BMI between 17.0 and 18.5 kg/m^2^ when the psychiatrist determined that clinical history or other physiological information supported this judgment. Participants from the HC group completed an online survey and were recruited via advertisements on social media sites, supplemented using a snowball sampling method.

The study complied with the Code of Ethics of the World Medical Association (Declaration of Helsinki) for experiments involving humans. It was approved by the Ethics Committee of the Faculty of Psychology and Education Sciences, University of Porto (Reference 2018/12-6), the Data Protection Unit of the University of Porto (Reference 4/2020), and the Health Ethics Committee of the University Hospital Center of São João (Reference CA-301/18). Individuals from both groups voluntarily participated in the study without receiving monetary or in-kind rewards, and informed consent was obtained from all participants.

### 2.4. Statistical Analyses

Data were analyzed using the IBM SPSS Statistics version 29. Missing data accounted for less than 10% of the main dataset. According to Bennett [55], this low percentage suggests that our statistical analyses are not prone to bias, and there was no replacement of missing values. To check for outliers, we used boxplots and z-scores. No significant outliers were identified. Descriptive analysis was performed to characterize the sample and the study variables. After assuring no severe deviations from the normal distribution, based on skewness and kurtosis below |3| and |10|, respectively [56], independent-sample *t*-tests were conducted to compare the AN group with the HC group based on the study variables (i.e., body appreciation, functionality appreciation, body responsiveness, difficulties in emotion regulation, psychological QoL, and depression, anxiety, and stress symptoms). Cohen’s [57] statistic was used to measure effect size: 0.20 = small effect, 0.50 = moderate effect, and 0.80 = large effect.

Bivariate correlations between all variables were conducted separately for the AN and HC groups. According to Cohen [57], Pearson r values of 0.10, 0.30, and 0.50 were used to demarcate small, medium, and large effects, respectively. Fisher’s r-to-z transformations were used to evaluate differences in correlation coefficients between each PBI facet and the five outcome variables within the AN and HC groups. Fisher’s r-to-z transformation was performed by using the online statistical computation website VassarStats [58].

Five separate multiple regression analyses with the enter method explored which PBI indices predicted difficulties in emotion regulation and psychological well-being (criterion variables) in the AN group. Multicollinearity was examined by inspecting the Variance Inflation Factors (VIFs), ensuring that the values were below 10 [59]. A significance level of 0.05 was considered with a 95% confidence interval.

## 3. Results

### 3.1. Comparative Analysis of Variables between AN and HC Groups

Independent-sample *t*-tests (Table 1) revealed significant differences between groups of high magnitude in all variables, except for the BRQ-Perceived Connection, which was moderate (*d* = −0.66). These results indicate that the AN group reported poorer PBI and psychological well-being, as well as more difficulties in regulating emotions, than the HC group.

### 3.2. Correlations between Positive Body Image, Psychological Well-Being, and Difficulties in Emotion Regulation in AN

As shown in Table 2, body appreciation was the only PBI dimension that correlated significantly with all study variables in both groups. In both groups, it was negatively correlated with emotion regulation difficulties and emotional symptoms (AN: −0.58 < *r* < −0.78; HC: −0.37 < *r* < −0.62) and positively correlated with psychological QoL (AN: r = 81; HC: r = 0.64). Regarding other PBI variables, results differed according to the group. In the AN group, functionality appreciation (|0.49| < *r* < |0.72|) and factors of body responsiveness (|0.36| < *r* < |0.61|) presented moderate-to-strong correlations with emotion regulation and indicators of psychological well-being. However, in the HC group, functionality appreciation and BRQ-Importance correlated with emotion regulation, depression, and psychological QoL (|0.30| < *r* < |0.52|) but not with anxiety and stress (*p* < 0.05). In the HC group, BRQ-Perceived Connection presented low correlations with the study variables (|0.11| < *r* < |0.26|). Lastly, emotion regulation difficulties and psychological QoL presented a negative, strong, and significant correlation both in the AN group (*r* = −0.74; *p* < 0.001) and the HC group (*r* = −0.59; *p* < 0.001).

Fisher’s r-to-z transformations indicated that body appreciation and functionality appreciation presented significantly different correlation coefficients with psychological well-being variables within the AN and HC groups (*p* < 0.05; higher correlations in the AN group). The only exception was the correlation between body appreciation and anxiety (z = −1.53, *p* = 0.063). Regarding body responsiveness, the only statistically significant differences observed were in the correlation coefficients related to the DASS. Specifically, significant differences were observed between BRQ-Importance and anxiety (*z* = −1.66, *p* = 0.049) and between BRQ-Perceived Connection and depression (*z* = −1.86, *p* = 0.031) and stress (*z* = −2.14, *p* = 0.016). The correlation coefficients between PBI variables and emotion regulation difficulties were not significantly different between AN and HC participants (*p* > 0.05). Detailed results are described in Table 3.

### 3.3. Multiple Regressions

To assess whether PBI facets (independent variables) predicted difficulties in emotion regulation, depression, anxiety, and stress symptoms, and psychological QoL (criterion variables), five separate multiple regression analyses were conducted. The VIFs for all regressions were <10, which did not suggest the possibility of multicollinearity. As displayed in Table 4, all models were significant, with the PBI variables accounting for 58% of the variance in the difficulties in emotion regulation, 62% in depression, 36% in anxiety, 45% in stress, and 72% in psychological QoL. Body appreciation is the PBI variable that makes the largest unique contribution to the prediction of emotion regulation difficulties, β = −0.55, *t* (4) = −3.81, *p* = < 0.001, depression, β = −0.62, *t* (4) = −3.71, *p* < 0.001, stress β = −0.53, *t* (4) = −2.60, *p* = 0.01, and psychological QoL, β = 0.60, *t* (4) = 5.21, *p* < 0.001. Body responsiveness was not a significant predictor. Functionality appreciation only emerged as a significant predictor of psychological QoL (β = 0.25, *t* (4) = 2.37, *p* < 0.02). PBI accounted for 36% of the variability of anxiety scores, but none of the specific PBI variables made a significant, unique contribution to the equation.

## 4. Discussion

Centering on body image as a fundamental aspect of AN, this study sought to comprehensively examine PBI in these patients, contributing to advancements in this unexplored field. We compared the PBI-related dimensions between individuals with AN and an HC group and investigated whether PBI predicted difficulties in emotion regulation, emotional symptoms (depression, anxiety, and stress), and psychological QoL. To the best of our knowledge, this is the first study that characterizes PBI in this eating disorder beyond body appreciation and analyzes its relationship with several facets of mental well-being. As anticipated, the AN group reported a lower appreciation for body appearance and functional abilities, as well as a tendency for reduced attunement to the body’s needs and use of embodied information to guide behavior, compared to the HC group. These findings contribute to a better understanding of some typical symptoms of AN. For instance, the excessive focus on weight, shape, and overall appearance limits the appreciation of other aspects of the body, such as its functionality. Low body awareness translates into difficulty interpreting bodily signals, hindering the adoption of adaptative self-care behaviors like intuitive eating and diminishing awareness of the impact of behaviors such as fasting, compulsive exercise, and purgative behaviors. Notably, the differences between AN and HC participants in PBI were of high magnitude. Taken together, these findings emphasize the pivotal role of PBI in understanding the body and mind’s experience within this eating disorder.

The relationship between PBI and well-being outcomes was supported in this study, echoing findings from previous investigations with non-clinical samples, e.g., [11,16,17,18]. In practice, the close link between these variables in the AN group suggests that PBI can be a protective mechanism for the co-occurrence of emotional disorders and a facilitator of QoL. It is also possible that the effect of PBI on psychological well-being could occur via emotion regulation. In other words, by enhancing PBI, a stronger body–mind connection is established, facilitating the accurate detection and interpretation of bodily cues [34]. This process can foster interoceptive awareness, an essential mechanism of emotion regulation [36,37]. With an increased capacity for adaptive emotion regulation, individuals with AN are less prone to developing emotional symptoms [29,30] and use food to deal with stressful situations [25]. This mediating relationship is a theoretical hypothesis, but we believe that substantial evidence supports its future examination using a longitudinal methodology. Our findings also strengthen this hypothesis, demonstrating a strong correlation between emotion regulation and both PBI and psychological well-being variables.

Given the multidimensional nature of PBI, this study offers insights into the distinct interaction of each PBI variable with psychological well-being in individuals with AN. Body appreciation and gratitude for bodily functions were strongly associated with emotional symptoms and psychological QoL, presenting correlation coefficients significantly higher in the AN group than in HCs. It was noteworthy that the negative relationship of functionality appreciation with anxiety and stress was strong in the AN group, while it was weak and not statistically significant in the HC group. The correlation coefficient with psychological QoL was also significantly higher in the AN group. These findings suggest that fostering functionality appreciation may be particularly relevant in the context of AN. The literature to date has indicated that overestimation of body size does not stem exclusively from visual misperception [60] and might also be driven by distorted attitudes regarding the desired body, such as considering underweight bodies attractive [61]. Thus, embracing a functional conceptualization of the body can alleviate internal and external sources of distress, encompassing concerns about self-image and fear of weight gain. Moreover, by appreciating the body’s capabilities in various domains, such as internal processes (e.g., bodily senses and sensations) and communication with others (e.g., via body language), individuals with AN are more likely to experience pleasure and greater satisfaction with themselves and their lives. Ultimately, valuing functionality may lead to enhancements in other aspects of PBI [62], in particular, body appreciation and the importance placed on bodily sensations for guidance (BRQ-Importance factor), as indicated by correlational analyses in this study. It is worth noting that body functionality is a valuable construct in relation to PBI [62] and it has gained increased importance as a means to overcome negative body image. For instance, it is proposed to be a core component of body neutrality—an emerging concept within the body image literature that encourages a non-judgmental attitude towards the body and prioritizes functionality over appearance. Unlike body positivity, which emphasizes that all bodies are beautiful, body neutrality removes the expectation of specific feelings toward the body, such as positivity or negativity. This different angle prioritizes what the body can do over how it looks [63] and can be a useful approach in eating disorder treatment [64].

Body responsiveness presented a positive and significant association with psychological QoL, with no significant differences between groups. Interestingly, a distinct pattern emerged in relation to depression, anxiety, and stress. Both BRQ factors correlated negatively and significantly with emotional symptoms, but only in the AN group (moderate-to-strong correlations). The findings suggest a specific role for body responsiveness in individuals with AN. This eating disorder is characterized by a fundamental conflict with embodiment, leading to patients experiencing confusion around bodily sensations and difficulties in considering their bodies as personal spaces [65]. In this context, body responsiveness could serve as a facilitator for connecting psychological and physical states, allowing for the recognition and regulation of inner states.

The overall contribution of PBI variables to predicting emotion regulation and psychological well-being in AN was remarkable, accounting for a percentage of variance ranging from 36% (anxiety) to 72% (psychological QoL). Among PBI variables, body appreciation was the best predictor; its inclusion enhanced all models’ ability to explain the outcome’s variations, except for anxiety. Furthermore, it was the only significant predictor in nearly all models. Only psychological QoL had another significant predictor: functionality appreciation. These findings hold important implications that warrant further discussion. Firstly, the hypothesis positing PBI as a catalyst for psychological health is reinforced by this study. As a whole, these PBI dimensions seem to create a favorable context for emotional stability in AN. In this process, the different aspects of PBI may enhance each other, but it is plausible that they make specific contributions, as discussed above. Secondly, the unique contribution of body appreciation as a predictor can be explained by the fact that this concept allows for a broader perspective of the experience of body image [45]. Although the BAS-2 does not incorporate all elements of PBI, it focuses on several core features, such as holding favorable opinions of the body, accepting the body despite perceived imperfections, and protecting the body by rejecting unrealistic societal appearance ideals. Thus, body appreciation can be a pivotal factor to consider in AN onset prevention, treatment, and relapse prevention. Thirdly, some implications for practice should be acknowledged. Emotional education has been regarded as a central aspect of AN treatment [28]. Building on our study, we advocate for expanding this approach to encompass mechanisms that also support the development of PBI, such as body and interoceptive awareness, body trust, and the body–mind connection. This approach can serve as the foundation for addressing other PBI-related aspects, including respecting and appreciating the body’s features, functions, and health.

### Limitations

While promising, these findings should be interpreted while considering some limitations. Data were collected exclusively through self-report measures, which are prone to social desirability. The results may not fully reflect the existent emotional and mental state of the participants. To offer a more comprehensive understanding of the mechanisms through which PBI can facilitate AN recovery, complementary qualitative research methods (e.g., interviews and focus groups) should be employed. It is important to note that the BRQ-Perceived Connection factor exhibited low internal consistency. While these Cronbach’s α values may be considered acceptable given the complexity of the construct and the limited number of items in this factor [66], findings pertaining to this factor should be interpreted with caution. The restriction to the female gender in our study sample and the recruitment of AN outpatients from a specific geographic region pose limitations on the generalizability of our results. To enhance the broader applicability of such findings, future research should prioritize the inclusion of more diverse samples in terms of demographics and social identity. For instance, it is known that body image disturbance affects individuals across gender and sexual orientation, but there seems to be some variability associated with these constructs [67]. In addition, the cross-sectional nature of this study did not allow for drawing causal inferences. Future research using prospective and longitudinal designs is recommended to provide valuable insights into the long-term effects of PBI on psychological well-being and emotion regulation in people with AN. Examining potential mediators, such as emotion regulation and global self-esteem, is also encouraged to better understand how PBI can affect mental health and eating behavior. Finally, it is important to note that this study does not include an AN symptom assessment, making it impossible to examine the direct effect of PBI on these symptoms. Thus, despite psychological well-being as a central criterion for eating disorder recovery [68], we cannot assume that PBI would be associated with a decrease in AN-related symptomatology. Exploring this question in future research would be intriguing. Hypothetically, fostering PBI could lead to mindful self-care and, consequently, a reduction in disordered eating behaviors.

## 5. Conclusions

This study emphasizes the close association of body appreciation, functionality appreciation, and body responsiveness with mental well-being in AN. Building on these results, we posit that fostering PBI may improve emotion regulation, reduce emotional symptoms, and diminish reliance on food for coping, thereby contributing to psychological well-being. Our findings suggest that a lifestyle emphasizing the mind–body connection has the potential to enhance well-being and promote healthy eating behaviors, making it a relevant element for both the treatment and prevention of AN.

## Figures and Tables

**Table 1:** Comparisons between the AN group and the HC group.

	AN Group (*n* = 64)		HC Group (*n* = 95)		*t*	*df*	Cohen’s *d*	95% CI		*p*
	*M*	*SD*	*M*	*SD*				Lower	Upper	
Body appreciation	2.49	0.75	3.67	0.66	−10.40	155	−1.70	−2.07	−1.33	<0.001
Functionality appreciation	3.54	0.82	4.36	0.49	−7.91	157	−1.28	−1.63	−0.93	<0.001
BRQ										
Importance	3.61	1.45	5.29	1.46	−7.13	157	−1.15	−1.49	−0.81	<0.001
Perceived Connection	3.10	1.39	4.17	1.74	−4.08	156	−0.66	−0.99	−0.34	<0.001
DERS Total	115.18	27.34	79.52	21.12	9.16	154	1.50	1.14	1.86	<0.001
DASS										
Depression	10.62	6.99	3.81	3.50	7.87	145	1.36	0.98	1.73	<0.001
Anxiety	7.77	6.34	3.72	3.38	5.06	145	0.87	0.52	1.23	<0.001
Stress	11.40	5.57	6.46	4.07	6.16	145	1.06	0.70	1.42	<0.001
Psychological QoL	11.62	2.61	14.74	1.98	−8.58	157	−1.39	−1.74	−1.03	<0.001

Note: CI = confidence interval; DERS Total = total score of the Difficulties Emotion Regulation Scale; BRQ = Body Responsiveness Questionnaire; DASS = Depression, Anxiety and Stress Scale; QoL = quality of life.

**Table 2:** Bivariate correlations between variables for the AN group in the top diagonal and HC group in the bottom diagonal.

	1	2	3	4	5	6	7	8	9
1. Body appreciation	-	0.69 ***	0.59 ***	0.58 ***	−0.75 ***	−0.78 ***	−0.58 ***	−0.65 ***	0.81 ***
2. Functionality appreciation	0.53 ***	-	0.58 ***	0.31 *	−0.60 ***	−0.63 ***	−0.50 ***	−0.49 ***	0.72 ***
3. BRQ—Importance	0.60 ***	0.53 ***	-	0.43 ***	−0.54 ***	−0.53 ***	−0.42 **	−0.40 **	0.61 ***
4. BRQ—Perceived Connection	0.24 *	0.09	0.14	-	−0.49 ***	−0.46 ***	−0.36 **	−0.46 ***	0.39 **
5. DERS Total	−0.62 ***	−0.40 ***	−0.43 ***	−0.26 **	-	0.80 ***	0.64 ***	0.76 ***	−0.74 ***
6. DASS—Depression	−0.53 ***	−0.30 **	−0.35 ***	−0.17	0.57 ***	-	0.82 ***	0.87 ***	−0.79 ***
7. DASS—Anxiety	−0.38 ***	−0.17	−0.16	−0.18	0.49 ***	0.69 ***	-	0.77 ***	−0.56 ***
8. DASS—Stress	−0.37 ***	−0.09	−0.20	−0.11	0.50 ***	0.72 ***	0.79 ***	-	−0.65 ***
9. Psychological QoL	0.64 ***	0.49 ***	0.52 ***	0.23 *	−0.59 ***	−0.60 ***	−0.38 ***	−0.33 ***	-

Note; AN group *n* = 64, HC group *n* = 95; DERS Total = total score of the Difficulties Emotion Regulation Scale; BRQ = Body Responsiveness Questionnaire; DASS = Depression, Anxiety and Stress Scale; QoL = quality of life. * *p* < 0.05; ** *p* < 0.01; *** *p* < 0.001.

**Table 3:** Fisher’s z transformation values and associated *p*-values for group comparisons of the correlation coefficients.

	DERS Total	DASS—Depression	DASS—Anxiety	DASS—Stress	Psychological QoL
Body appreciation	−1.39	−2.56	−1.53	−2.20	2.17
	0.082	0.005	0.063	0.014	0.015
Body functionality	−1.60	−2.45	−2.10	−2.52	2.21
	0.055	0.007	0.018	0.006	0.013
BRQ—Importance	−0.86	−1.27	−1.66	−1.27	0.85
	0.196	0.102	0.049	0.102	0.199
BRQ—Perceived Connection	−1.57	−1.86	−1.11	−2.14	1.09
	0.058	0.031	0.133	0.016	0.138

Note: AN group *n* = 64, HC group *n* = 95; DERS Total = total score of the Difficulties Emotion Regulation Scale; BRQ = Body Responsiveness Questionnaire; DASS = Depression, Anxiety and Stress Scale; QoL = quality of life. *p* < 0.05; *p* < 0.01; *p* < 0.001.

**Table 4:** Results of the multiple regressions with total score of the Difficulties in Emotion Regulation Scale, depression, anxiety, and stress symptoms, and psychological QoL as the criterion variables for the AN group (*n* = 64).

	B	95% CI for B		SE B	β	*t*	*p*	R^2^	Adj R^2^
		LL	UL						
DERS Total									
Body appreciation	−20.14	−30.73	−9.54	5.28	−0.55	−3.81	<0.001		
Functionality appreciation	−6.17	−15.06	2.72	4.43	−0.18	−1.39	0.17		
BRQ—Importance	−0.49	−5.18	4.21	2.34	−0.03	−0.21	0.84		
BRQ—Perceived Connection	−1.93	−6.28	2.42	2.17	−0.10	−0.89	0.38		
*F* (4, 54) = 18.84, *p* < 0.001								0.58	0.55
DASS—Depression									
Body appreciation	−5.73	−8.85	−2.62	1.55	−0.62	−3.71	<0.001		
Functionality appreciation	−1.12	−3.47	1.22	1.16	−0.14	−0.97	0.34		
BRQ—Importance	−0.25	−1.34	0.84	0.54	−0.05	−0.46	0.65		
BRQ—Perceived Connection	−0.30	−1.50	0.91	0.60	−0.06	−0.49	0.62		
*F* (4, 45) = 18.48, *p* < 0.001								0.62	0.59
DASS—Anxiety									
Body appreciation	−3.20	−6.85	0.45	1.81	−0.38	−1.77	0.08		
Functionality appreciation	−1.12	−3.87	1.62	1.36	−0.15	−0.82	0.41		
BRQ—Importance	−0.32	−1.60	0.96	0.64	−0.08	−0.51	0.61		
BRQ—Perceived Connection	−0.36	−1.77	1.05	0.70	−0.08	−0.51	0.61		
*F* (4, 45) = 6.25, *p* < 0.001								0.36	0.30
DASS—Stress									
Body appreciation	−3.86	−6.85	−0.87	1.49	−0.53	−2.60	0.01		
Functionality appreciation	−0.42	−2.67	1.83	1.12	−0.06	−0.37	0.71		
BRQ—Importance	0.02	−1.03	1.07	0.52	0.01	0.04	0.97		
BRQ—Perceived Connection	−0.60	−1.75	0.56	0.57	−0.15	−1.04	0.31		
*F* (4, 45) = 8.77, *p* < 0.001								0.45	0.41
Psychological QoL									
Body appreciation	2.11	1.30	2.93	0.41	0.60	5.21	<0.001		
Functionality appreciation	0.79	0.12	1.45	0.33	0.25	2.37	0.02		
BRQ—Importance	0.28	−0.05	0.61	0.17	0.16	1.70	0.10		
BRQ—Perceived Connection	−0.19	−0.52	0.15	0.17	−0.10	−1.11	0.27		
*F* (4, 56) = 35.86, *p* < 0.001								0.72	0.70

Note: B = unstandardized coefficient; CI = confidence interval; SE B = standard error for the unstandardized coefficient; β = standardized coefficient; R^2^ = R square; Adj R^2^ = Adjusted R square; LL = lower limit; UL = upper limit; DERS Total = total score of the Difficulties Emotion Regulation Scale; DASS = Depression, Anxiety and Stress Scale; QoL = quality of life; BRQ = Body Responsiveness Questionnaire.

## Data Availability

The data presented in this study are available on request from the corresponding author. The data are not publicly available due to the inclusion of information that could compromise the privacy of the research participants.

## References

[1] American Psychiatric Association (2022). Diagnostic and Statistical Manual of Mental Disorders.

[2] Boehm I., Finke B., Tam F.I., Fittig E., Scholz M., Gantchev K., Roessner V., Ehrlich S. (2016). Effects of perceptual body image distortion and early weight gain on long-term outcome of adolescent anorexia nervosa. Eur. Child Adolesc. Psychiatry.

[3] Calugi S., El Ghoch M., Conti M., Dalle Grave R. (2018). Preoccupation with shape or weight, fear of weight gain, feeling fat and treatment outcomes in patients with anorexia nervosa: A longitudinal study. Behav. Res. Ther..

[4] Sattler F.A., Eickmeyer S., Eisenkolb J. (2020). Body image disturbance in children and adolescents with anorexia nervosa and bulimia nervosa: A systematic review. Eat. Weight Disord..

[5] Glashouwer K.A., van der Veer R.M.L., Adipatria F., de Jong P.J., Vocks S. (2019). The role of body image disturbance in the onset, maintenance, and relapse of anorexia nervosa: A systematic review. Clin. Psychol. Rev..

[6] Cash T.F., Cash T.F., Pruzinsky T. (2002). Cognitive-behavioral perspectives on body image. Body Image: A Handbook of Theory, Research, and Clinical Practice.

[7] Grogan S. (2016). Body Image: Understanding Body Dissatisfaction in Men, Women and Children.

[8] Tylka T.L., Wood-Barcalow N.L. (2015). What is and what is not positive body image? Conceptual foundations and construct definition. Body Image.

[9] Avalos L., Tylka T.L., Wood-Barcalow N. (2005). The Body Appreciation Scale: Development and psychometric evaluation. Body Image.

[10] Baceviciene M., Jankauskiene R. (2020). Associations between body appreciation and disordered eating in a large sample of adolescents. Nutrients.

[11] Gillen M.M. (2015). Associations between positive body image and indicators of men’s and women’s mental and physical health. Body Image.

[12] Tylka T.L., Kroon Van Diest A.M. (2013). The Intuitive Eating Scale-2: Item refinement and psychometric evaluation with college women and men. J. Couns. Psychol..

[13] Tylka T.L., Homan K.J. (2015). Exercise motives and positive body image in physically active college women and men: Exploring an expanded acceptance model of intuitive eating. Body Image.

[14] Linardon J., Tylka T.L., Burnette C.B., Shatte A., Fuller-Tyszkiewicz M. (2022). Understanding the role of positive body image during digital interventions for eating disorders: Secondary analyses of a randomized controlled trial. Body Image.

[15] Koller K.A., Thompson K.A., Miller A.J., Walsh E.C., Bardone-Cone A.M. (2020). Body appreciation and intuitive eating in eating disorder recovery. Int. J. Eat. Disord..

[16] Winter V.R., Gillen M.M., Cahill L., Jones A., Ward M. (2019). Body appreciation, anxiety, and depression among a racially diverse sample of women. J. Health Psychol..

[17] Lemoine J.E., Konradsen H., Lunde Jensen A., Roland-Lévy C., Ny P., Khalaf A., Torres S. (2018). Factor structure and psychometric properties of the Body Appreciation Scale-2 among adolescents and young adults in Danish, Portuguese, and Swedish. Body Image.

[18] O’Neill E.A., Ramseyer Winter V., Pevehouse D. (2018). Exploring body appreciation and women’s health-related quality of life: The moderating role of age. J. Health Psychol..

[19] Oldershaw A., Hambrook D., Stahl D., Tchanturia K., Treasure J., Schmidt U. (2011). The socio-emotional processing stream in anorexia nervosa. Neurosci. Biobehav. Rev..

[20] Oldershaw A., Lavender T., Sallis H., Stahl D., Schmidt U. (2015). Emotion generation and regulation in anorexia nervosa: A systematic review and meta-analysis of self-report data. Clin. Psychol. Rev..

[21] Torres S., Guerra M.P., Lencastre L., Roma-Torres A., Brandão I., Queirós C., Vieira F. (2011). Cognitive processing of emotions in anorexia nervosa. Eur. Eat. Disord. Rev..

[22] Westwood H., Kerr-Gaffney J., Stahl D., Tchanturia K. (2017). Alexithymia in eating disorders: Systematic review and meta-analyses of studies using the Toronto Alexithymia Scale. J. Psychosom. Res..

[23] Aldao A., Nolen-Hoeksema S., Schweizer S. (2010). Emotion-regulation strategies across psychopathology: A meta-analytic review. Clin. Psychol. Rev..

[24] Hatch A., Madden S., Kohn M., Clarke S., Touyz S., Williams L.M. (2010). Anorexia nervosa: Towards an integrative neuroscience model. Eur. Eat. Disord. Rev..

[25] Haynos A.F., Fruzzetti A.E. (2011). Anorexia nervosa as a disorder of emotion dysregulation: Evidence and treatment implications. Clin. Psychol..

[26] Ribeiro A., Sinval J., Félix S., Guimarães C., Machado B.C., Gonçalves S., de Lourdes M., Conceição E.M. (2023). Food addiction and grazing—The role of difficulties in emotion regulation and negative urgency in university students. Nutrients.

[27] Fairburn C.G., Cooper Z., Shafran R. (2003). Cognitive behaviour therapy for eating disorders: A “transdiagnostic” theory and treatment. Behav. Res. Ther..

[28] Castro T.F., Miller K., Araújo M.X., Brandão I., Torres S. (2021). Emotional processing in recovered anorexia nervosa patients: A 15 year longitudinal study. Eur. Eat. Disord. Rev..

[29] Lulé D., Schulze U.M.E., Bauer K., Schöll F., Müller S., Fladung A.-K., Uttner I. (2014). Anorexia nervosa and its relation to depression, anxiety, alexithymia and emotional processing deficits. Eat. Weight Disord..

[30] Torres S., Guerra M.P., Lencastre L., Miller K., Vieira F.M., Roma-Torres A., Brandão I., Costa P. (2015). Alexithymia in anorexia nervosa: The mediating role of depression. Psychiatry Res..

[31] Khodabakhsh M.R., Borjali A., Sohrabi F., Farrokhi N.A.F. (2015). The role of emotion regulation difficulties as a mediator of the relationship between body image disturbance and disordered eating behavior. Int. J. Pediatr..

[32] Linardon J., McClure Z., Tylka T.L., Fuller-Tyszkiewicz M. (2022). Body appreciation and its psychological correlates: A systematic review and meta-analysis. Body Image.

[33] Longhurst P., Swami V. (2023). A feeling difficult to identify: Alexithymia is inversely associated with positive body image in adults from the United Kingdom. J. Affect. Disord..

[34] Torres S., Vieira A.I., Vieira F.M., Lencastre L., Guerra M.P., Miller K.M., Barbosa M.R. (2023). Psychometric analysis of the Body Responsiveness Questionnaire in the Portuguese population. Sci. Rep..

[35] Daubenmier J.J. (2005). The relationship of yoga, body awareness, and body responsiveness to self-objectification and disordered eating. Psychol. Women Q..

[36] Füstös J., Gramann K., Herbert B.M., Pollatos O. (2012). On the embodiment of emotion regulation: Interoceptive awareness facilitates reappraisal. Soc. Cogn. Affect. Neurosci..

[37] Price C.J., Hooven C. (2018). Interoceptive awareness skills for emotion regulation: Theory and approach of mindful awareness in body-oriented therapy (MABT). Front. Psychol..

[38] Munroe M. (2022). Positive embodiment for wellbeing researchers and practitioners: A narrative review of emerging constructs, measurement tools, implications, and future directions. Int. J. Wellbeing.

[39] Burychka D., Miragall M., Baños R.M. (2021). Towards a comprehensive understanding of body image: Integrating positive body image, embodiment and self-compassion. Psychol. Belg..

[40] Cash T.F., Cash T.F., Smolak L. (2011). Cognitive-behavioral perspectives on body image. Body Image: A Handbook of Science, Practice, and Prevention.

[41] Webb J.B., Butler-Ajibade P., Robinson S.A. (2014). Considering an affect regulation framework for examining the association between body dissatisfaction and positive body image in Black older adolescent females: Does body mass index matter?. Body Image.

[42] Fuchs T. (2022). The disappearing body: Anorexia as a conflict of embodiment. Eat. Weight Disord..

[43] Laporta-Herrero I., Jáuregui-Lobera I., Serrano-Troncoso E., Garcia-Argibay M., Cortijo-Alcarria M.C., Santed-Germán M.-A. (2022). Attachment, body appreciation, and body image quality of life in adolescents with eating disorders. Eat. Disord..

[44] American Psychiatric Association (2013). Diagnostic and Statistical Manual of Mental Disorders.

[45] Tylka T.L., Wood-Barcalow N.L. (2015). The Body Appreciation Scale-2: Item refinement and psychometric evaluation. Body Image.

[46] Meneses L., Torres S., Miller K.M., Barbosa M.R. (2019). Extending the use of the Body Appreciation Scale-2 in older adults: A Portuguese validation study. Body Image.

[47] Alleva J.M., Tylka T.L., Kroon Van Diest A.M. (2017). The Functionality Appreciation Scale (FAS): Development and psychometric evaluation in U.S. community women and men. Body Image.

[48] Marta-Simões J., Oliveira S., Ferreira C. The Portuguese validation of the Functionality Appreciation Scale. Proceedings of the IV International Congress CINEICC.

[49] Gratz K.L., Roemer L. (2004). Multidimensional assessment of emotion regulation and dysregulation: Development, factor structure, and initial validation of the Difficulties in Emotion Regulation Scale. J. Psychopathol. Behav. Assess..

[50] Coutinho J., Ribeiro E., Ferreirinha R., Dias P. (2010). Versão portuguesa da Escala de Dificuldades de Regulação Emocional e sua relação com sintomas psicopatológicos. Arch. Clin. Psychiatry.

[51] Lovibond P.F., Lovibond S.H. (1995). The structure of negative emotional states: Comparison of the Depression Anxiety Stress Scales (DASS) with the Beck Depression and Anxiety Inventories. Behav. Res. Ther..

[52] Pais-Ribeiro J.L., Honrado A., Leal I. (2004). Contribuição para o estudo da adaptação portuguesa das Escalas de Ansiedade, Depressão e Stress (EADS) de 21 itens de Lovibond e Lovibond. Psicol. Saúde Doenças.

[53] WHOQOL Group (1998). Development of the World Health Organization WHOQOL-BREF Quality of Life Assessment. Psychol. Med..

[54] Vaz Serra A., Canavarro M.C., Simões M., Pereira M., Gameiro S., Quartilho M.J., Rijo D., Paredes C., Paredes T. (2006). Estudos psicométricos do instrumento de avaliação da qualidade de vida da Organização Mundial de Saúde (WHOQOL-Bref) para Português de Portugal. Psiquiatr. Clínica.

[55] Bennett D.A. (2001). How can I deal with missing data in my study?. Aust. N. Z. J. Public Health.

[56] Kline R.B. (2011). Principles and Practice of Structural Equation Modeling.

[57] Cohen J. (1988). Statistical Power Analysis for the Behavioral Sciences.

[58] Lowry R. (2001). VassarStats: Website for Statistical Computation—Significance of the Difference between Two Correlation Coefficients. http://vassarstats.net/rdiff.html.

[59] Hair J.F., Anderson R.E., Tatham R.L., Black W.C. (1995). Multivariate Data Analysis.

[60] Gadsby S., Zopf R., Brooks K.R., Schumann A., de la Cruz F., Rieger K., Murr J., Wutzler U., Bär K.-J. (2023). Testing visual self-misperception in anorexia nervosa using a symmetrical body size estimation paradigm. Int. J. Eat. Disord..

[61] Mölbert S.C., Thaler A., Mohler B.J., Streuber S., Romero J., Black M.J., Zipfel S., Karnath H.O., Giel K.E. (2018). Assessing body image in anorexia nervosa using biometric self-avatars in virtual reality: Attitudinal components rather than visual body size estimation are distorted. Psychol. Med..

[62] Alleva J.M., Tylka T.L. (2021). Body functionality: A review of the literature. Body Image.

[63] Pellizzer M.L., Wade T.D. (2023). Developing a definition of body neutrality and strategies for an intervention. Body Image.

[64] Perry M., Watson L., Hayden L., Inwards-Breland D. (2019). Using body neutrality to inform eating disorder management in a gender diverse world. Lancet Child Adolesc. Health.

[65] Cascino G., Castellini G., Stanghellini G., Ricca V., Cassioli E., Ruzzi V., Monteleone P., Monteleone A.M. (2019). The role of the embodiment disturbance in the anorexia nervosa psychopathology: A network analysis study. Brain Sci..

[66] DeVellis R.F. (2016). Scale Development: Theory and Applications.

[67] Dahlenburg S.C., Gleaves D.H., Hutchinson A.D., Coro D.G. (2020). Body image disturbance and sexual orientation: An updated systematic review and meta-analysis. Body Image.

[68] de Vos J.A., LaMarre A., Radstaak M., Bijkerk C.A., Bohlmeijer E.T., Westerhof G.J. (2017). Identifying fundamental criteria for eating disorder recovery: A systematic review and qualitative meta-analysis. J. Eat. Disord..

